# Take the First-Person Perspective to Become Dementia-Friendly: The Use of 360° Video for Experiencing Everyday-Life Challenges With Cognitive Decline

**DOI:** 10.3389/fpsyg.2020.01117

**Published:** 2020-06-30

**Authors:** Francesca Morganti, Nicola Palena, Paola Savoldelli, Andrea Greco

**Affiliations:** Department of Human and Social Sciences, University of Bergamo, Bergamo, Italy

**Keywords:** Dementia-Friendly Community, dementia needs, ViveDe, 360° video, first-person experience

## Abstract

The current spread of dementia is engendering an emergency that is not limited to the medical issues but also involves its social dimension. Accordingly, it is necessary to promote a perspective change about the disease that supports a more inclusive view of people with dementia. To ensure this, Dementia-Friendly Communities (DFCs) have recently been developed. Nonetheless, it is not always effortless to deal with people with dementia in an inclusive way because of misconceptions about how they perceive everyday contexts and react in everyday situations. We asked 170 individuals (aged between 13 and 75) to “put themselves in the shoes of a person with dementia” for a few minutes, facilitating this through the use of a 360° video, and to try to experience how activities such as going shopping feel from the first-person perspective. Before and after the experience, participants expressed their opinions about the needs and the autonomies that are deemed to be granted to a person with dementia. The results revealed changes to social perspective after having experienced firsthand what living with dementia could be like. A deeper comprehension of what it is like to live with dementia appeared to be gained, and participants’ beliefs about the needs and daily autonomies of those with dementia were modified after the experience. It is possible to conclude that, through the change of perspective, people are more willing to be inclusive toward people with dementia, as is wished for in the DFC approach, although a wider formative intervention on how to be really inclusive still seems to be required.

## Introduction

The latest research developments on dementia have shown that there are still no unequivocal data about the causes of this disease (Kapasi et al., 2017). Nor is there an efficacious therapy to stem the cognitive impairments and psychological alterations that the various forms of dementia involve (Watt et al., 2019). Therefore, person-centered approaches (Kitwood, 1997), both for diagnosis and treatment, still seem to be among the most valuable solutions for people with dementia. Moreover, it is necessary to consider that, to date, one of the arrangements that is most effective in dementia treatment is to maintain a high quality of life for people already suffering from this illness (Landeiro et al., 2018).

Consequently, in parallel with research on maximizing the effects of prevention strategies (Hodes et al., 2019) and to support an early diagnosis (Paulsen et al., 2013), Dementia-Friendly Communities (DFC) are springing up all over the world (Alzheimer’s Disease International, 2016; Lin, 2016) Dementia-Friendly Communities are communities of citizens, not exclusively personally involved in dementia healthcare and/or relatives to people suffering from dementia, which promote inclusive lifestyles to people affected by this disease. Their main goal is to maximize the autonomy of the person with dementia within the urban context (Smebye et al., 2016) through improving their quality of life, extending their residence at home as far as possible, and maximizing their network of social relations. Worldwide DFCs are proposing educational projects in schools, organizations, and groups of individuals in order to support them in understanding, respecting, and supporting people who live with dementia. The development of DFCs tends to counteract, above all, the institutionalization of people who still have a less severe form of dementia (not such as to completely degrade their daily self-government), minimizing the effort required to ensure that their essential needs are met, which is often delegated too early to a caregiver. Dementia-Friendly Communities are connected in an international DFC network, even though each country and each specific community finds its own way to becoming Dementia-Friendly. The main cornerstones of DFCs are to remove obstacles to inclusion in society, to prevent stigma and fear about dementia in the general population, and to avoid under-estimation of the capabilities of people with dementia by professionals, stakeholders, and any community member.

Despite this effort, the concrete actions that support the community of citizens in becoming Dementia-Friendly clash daily with the misconception of dementia (Swaffer, 2014), which finds its prototypical representation in “ageism” (thinking that dementia is a pathology exclusive to elderly people), in “nihilism” (thinking that it is not possible to do anything for people with dementia if you are not a professional), and also in “ignorance” (thinking that dementia destroys the ability to understand the environment and to have goal-directed behaviors from its first diagnosis). In addition, the main resistance to becoming truly inclusive toward a person with dementia comes from the difficulty of understanding from a third-person perspective what underlies the observable unusual behaviors of a person with dementia within that person (these behaviors are often interpreted as not dependent on the disease). In particular, because dementia mainly affects the person’s cognitive and emotional capacities, which by their nature are not detectable by an external observer (Zahavi, 2008), people tend to not fully understand some behaviors a person with dementia generally shows (such as, for example, time-space disorientation or mood alteration) during a daily relational situation (such as encountering a casual acquaintance or taking part in a meeting).

To overcome these issues, approaches that encourage a change of perspective have recently been adopted. For example, the Virtual Dementia Tour^®^ has been widespread in the United States for many years; this is mainly aimed at caregivers and family members of people with dementia, showing them how some sensory and motor limitations can compromise the ability to solve simple problems (Beville, 2002). Though there are some doubts as to its evidence-based efficacy (Merizzi, 2018), the results of related studies show that there might be a change in the management of the patient by the caregivers after this experience.

With the objective of providing the general public with the opportunity to experience dementia from the inside through the use of new technologies, in Italy, the ViveDe project was developed by the Dementia-Friendly research group at the University of Bergamo^1^. The main goal of the project is to use virtual reality eyeglasses to present several everyday situations that people with dementia can face in everyday life through the use of 360° videos that are explorable on the *x*/*y* axis. Thus, even a member of the public who is not familiar with dementia has the possibility to take a first-person perspective on dementia during a daily activity and to experience how living with dementia might be (Morganti, 2019).

In this research, we aim at investigating if the ViveDe approach is useful for paving the way to becoming Dementia-Friendly. Our main hypothesis is that, after the ViveDe experience, which forces them to assume the first-person point of view, participants will change their social perspective on dementia, abandoning the stigma and reflecting on the role that they might have in promoting the autonomy of people living with dementia. Specifically, our research hypothesis will be that, after the experience with the immersive 360° video, the participant will modify their opinions about:

1. What the prerogatives of people with dementia are:
  (a) The need for continuous assistances after the dementia diagnosis.
  (b) The possibility of having a large amount of autonomy in daily activities.
  (c) The exclusive role of professionals in supporting dementia people.
2. What the demands of people with dementia are, in terms of those expressed by Maslow (1943):
  (a) To have basic needs (physiological and safety) granted.
  (b) To have psychological needs (social belonging and esteem) granted.
  (c) To have self-actualization needs granted.
3. What the possibilities of becoming Dementia-Friendly are through
  (a) Improved knowledge about dementia.
  (b) Modification of the perceived difficulty of being a friend to a person with dementia.

## Methods

### Participants

All participants were volunteers attending a public event in which the VideDe project was presented.

In total, 170 (65 males and 105 females) took part in the experiment, with ages ranging from 13 to 76 years (*M* = 39.29, SD = 17.78). Additionally, 99 participants declared that no relative of theirs was diagnosed with any form of dementia, whereas the remaining 71 did have such a relative.

### Procedure

**FIGURE 1:**
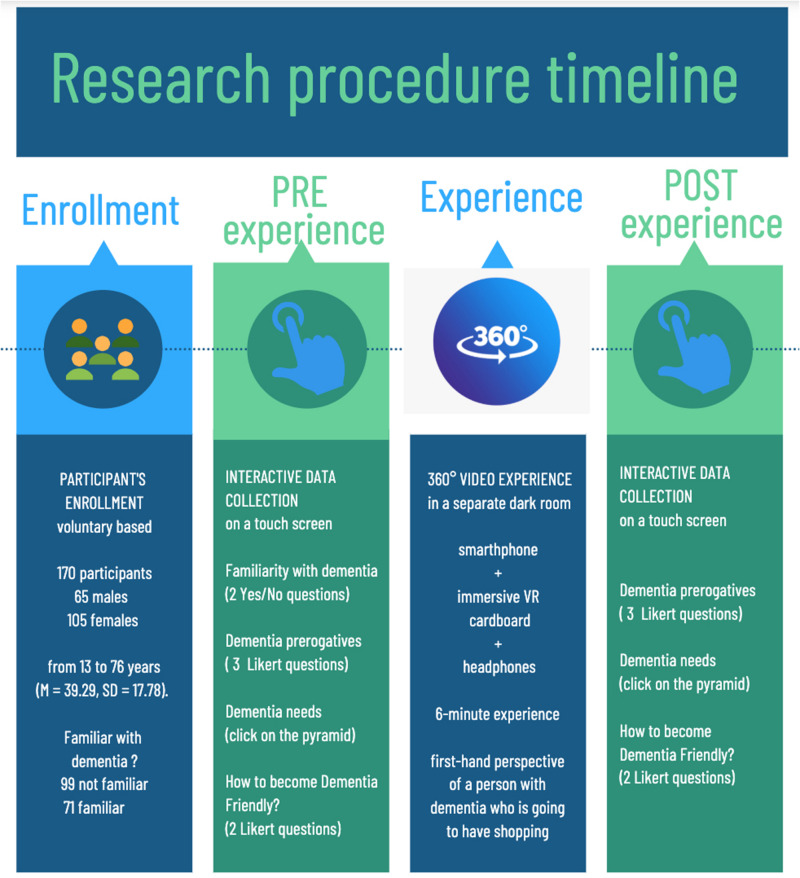
Research procedure timeline.

After having signed an informed consent form, participants entered the experimental setting and were guided by an experimenter to answer the questions described in the following paragraph, which were depicted on a touch-screen device. Participants were then taken into a separate room, where a brief video on how to interact with a 360° video was presented. After this explanation, participants could start their immersive experience with a 6-min ViveDe video under the supervision of one experimenter. Once the virtual experience was over, participants were again requested to provide answers to the same questions as were provided before the experience on the touch screen. The entire procedure (including the informed consent phase) took approximately 20 minutes. The experiment timeline is depicted in Figure 1.

### Interactive Data Collection

Data were collected in an anonymous form through the use of an interactive table placed at the entrance of the experimental setting. By clicking on the touch screen, participants provide to the experimenter information about their age and familiarity with dementia (Do they work with dementia? Are they relatives of people with dementia?).

Moreover, in order to explore the research hypothesis, on the interactive table participants have to:

1. Answer three questions to provide their opinions about the prerogatives of people with dementia. The questions are answered on a five-point Likert scale. The questions are about:
  (a) Assistance: Do you think that people with dementia need to have continuous familiar assistance during everyday activities?
  (b) Autonomy: Do you think that people can continue to autonomously meet their personal needs (such as going out to purchase goods or doing their housekeeping) just after a dementia diagnosis?
  (c) Institutionalization: Do you think people with dementia have to immediately ask for support from institutional welfare (such as nursing homes and/or professional caregivers) after their first diagnosis?
2. Click on a pyramid image that represents the five levels of individual needs defined by Maslow (1943) in order to answer the question, “Which needs do you consider as essential to be warranted to a person with dementia?” The needs depicted are (from the bottom to the top of the pyramid): physiological, safety, social belonging, esteem, and self-actualization. A brief description of the needs according to Maslow’s definitions is provided on the screen in order to avoid misunderstanding.
3. Answer two questions to provide their opinion about the perceived difficulty of becoming Dementia Friendly, in particular in terms of taking care of people diagnosed with dementia (“Please indicate on the depicted line a point that corresponds to how demanding you think it is to live with a person with dementia”) and in terms of the knowledge on dementia they believe they have (“Please indicate on the depicted line a point that corresponds to your knowledge on what dementia is”). The questions are answered on a 10-point Likert scale (1 = minimum, 10 = maximum).

### The Immersive Experience

**FIGURE 2:**
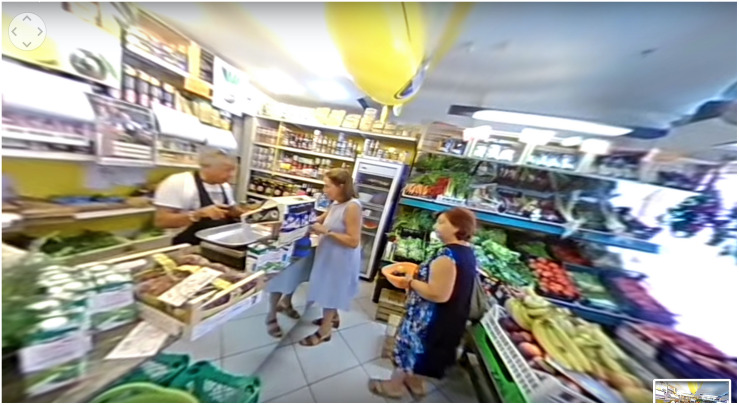
A ViveDe 360° video snapshot.

After the touch-screen phase, participants were conducted to a separate dark room within which they had the ViveDe experience. The experience was made possible through the use of the Homido virtual reality Headset V2 (a commercial device for smartphones with 100° FOV lenses, farsightedness and nearsightedness settings, and IPD and immersion adjustment^2^) and a set of headphones. Participants immersively experienced a 360° video downloadable on their smartphone from the www.vivede.it website. The video provides a six-minute experience of a person with dementia doing their daily shopping at the neighborhood bakery and greengrocer. In the video, a typical daily situation is represented (e.g., other customers at the same store being in a hurry while several cognitive impairments are experienced by the person with dementia in managing money or remembering the list of goods that have to be bought). A video snapshot is provided in Figure 2; the Italian version of the video is available at https://youtu.be/A15h8_UHWE4.

The 360° video is fully explorable on the *x*/*y* axis. The video provided to participants is from a first-person perspective (i.e., participants can perceive the entire scene as if they are in the shoes of a person with dementia). Moreover, the participants can hear firsthand the voice of a person with dementia as if it is their “thoughts.” The video, like the others developed in the ViveDe project, is the upshot of a previous research phase in which people with dementia, caregivers, and urban communities were involved in providing qualitative and quantitative information about how difficult is it to face everyday challenges when living with dementia (Morganti, 2019).

### Design

**FIGURE 3:**
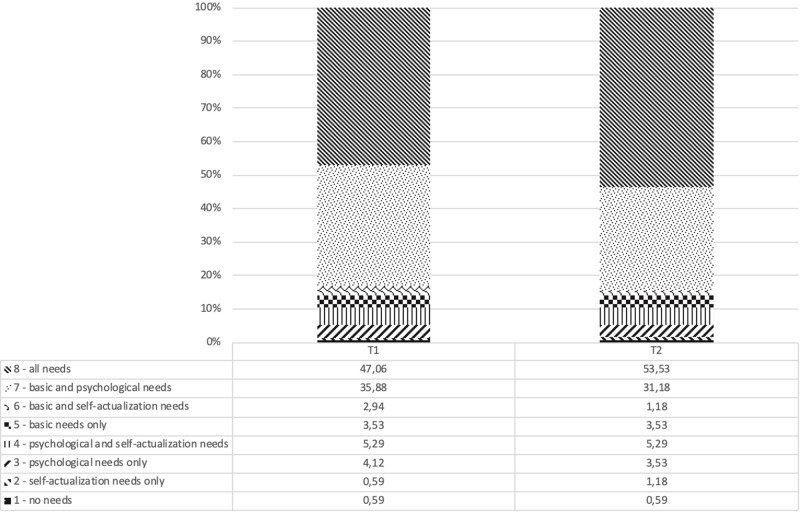
Relative percentage of the eight clusters at T1 and T2.

The only factor was “Time.” All participants filled the questions before and after the ViveDe experience. The dependent variables were:

(a) Prerogatives of people with dementia. Three questions measured on a five-point Likert scale (1 = not agree, 2 = slightly agree, 3 = partially agree, 4 = mainly agree, and 5 = totally agree) exploring the perceived need for assistance in dementia patients, the perceived right for autonomy, and the perceived need to be relocated to specialist health and social structures.
(b) Demands of people with dementia. The eight clusters reported in Figure 3 originated from the perceived need for each of the five levels of Maslow’s pyramid (physiological, safety, belonging, esteem, and self-actualization), which the participant either selected or not as being required by people with dementia;
(c) Possibility of becoming Dementia-Friendly. The perceived knowledge of the respondent about what dementia is and their perception of the difficulty of living with a person diagnosed with dementia, on a Likert scale ranging from 1 to 10, with no specific label.

## Data Analysis

The hypotheses concerning participants’ opinions about the prerogatives of people with dementia and participants’ opinions about the perceived difficulty of becoming Dementia-Friendly were explored by using SPSS 22 statistical software.

To explore participants’ opinions about the individual demands of people with dementia, analyses were conducted using Sleipner 2.1, which is a statistical package used for typological analyses and for studying individual development, that is, stability vs. change (Bergman and El-Khouri, 2002). An individual in one cluster is said to be “stable” if s/he shows a tendency to re-emerge in a similar cluster at a later time; on the contrary, “change” refers to a tendency to re-emerge in a different cluster. According to Bergman et al. (2003), individual stability is related, but not equivalent to, structural stability. In the present case, individual stability was evaluated by performing Fisher’s hypergeometric distribution test in single cells in the cross-tabulation of eight possible clusters at Time 1 (T1, pre-experience) and Time 2 (T2, post-experience). These clusters were obtained as follows.

The five levels of Maslow’s pyramid of needs were grouped into three possible levels: a base level (basic needs) including physiological needs and safety, a second level (psychological needs) including social belonging and esteem, and a third level (self-actualization needs) focusing on self-actualization only (Maslow, 1943, 1954). If a participant selected at least one of the two variables of the basic needs level (physiological needs and safety), it was concluded that such a participant believed that the basic needs were believed to be an important need for a person with dementia. Code “2” was assigned in this case. On the other hand, if none of the two needs was selected, it was believed that the participant believed that the basic needs were not relevant for people with dementia, and code “1” was employed. The same applied for psychological needs. Concerning the third level, which focuses on self-actualization, it was assessed only whether the participant reported that people with dementia have (coded as 2) or do not have (coded as 1) a need for self-actualization. Consequently, eight possible configurations/clusters were possible, which are reported in Figure 3.

The analysis was conducted using the EXACON module of Sleipner, which produces a contingency table for two categorical variables, in our case, cluster membership at T1 and T2. The EXACON procedure focuses on cell-wise analysis of types based on exact tests. Specifically, a type is said to occur in a cell if the observed frequency is much larger than the expected frequency and the associated hypergeometric probability is low; that is, we observe a significantly larger frequency than we expect to observe by chance alone. In the opposite case, an antitype is said to occur. In other words, this analysis evaluates the T1-to-T2 sequences of clusters for perceived needs to verify whether these sequences occur differently than expected by chance.

## Results

### Prerogatives of People With Dementia

Before running any analysis, correlations among the three questions were evaluated. As Table 1 shows, there were several significant correlations, suggesting that answers to one question have a relationship with answers to other questions. For this reason, a MANOVA was conducted with Time (T1, pre-experience vs. T2, post-experience) as the factor and answers to questions 1–3 (assistance, autonomy, and institutionalization) as dependent variables. There was a significant multivariate effect, *F*(3, 167), Wilks’ λ = 14.10, *p* < 0.001. At a univariate level, the effect for the question concerning assistance was not significant, *F*(1, 169) = 2.77, *p* = 0.09, partial η^2^ = 0.02. On the contrary, the effect was significant for autonomy, *F*(1, 169) = 34.65, *p* < 0.001, partial η^2^ = 0.17, and for institutionalization, *F*(1, 169) = 11.25, *p* < 0.01, partial η^2^ = 0.06. As Table 1 shows, participants reported similar levels of perceived need for assistance at T1 and T2, whereas they reported lower needs for autonomy and institutionalization at T2 compared to T1.

**TABLE 1:** Descriptives for answers of the three questions on assistance, autonomy and institutionalization and correlations among answers to the three questions.

	***M* (SD) pre-experience**	***M* (SD) post-experience**	***t*-test (169)**	**Assistance post-experience**	**Autonomy post-experience**	**Institutionalization post-experience**
Assistance pre-experience	3.68 (0.76)	3.82 (0.87)	−1.66	–	−0.58**	0.10
Autonomy pre-experience	2.86 (0.92)	2.34 (0.88)	5.89***	−0.16*		−0.11
Institutionalization pre-experience	3.39 (0.94)	3.06 (1.15)	3.35**	−0.33**	−0.06	–

The correlations under the diagonal are for the pre-experience answers and those in the upper triangle are for the post-experience answers. *p < 0.05, **p < 0.01, **p < 0.001.

### Demands of People With Dementia

Figure 3 reports the relative percentage of the eight clusters at T1 and T2. The analyses showed that there were 12 significant cells. Of these, four cells revealed that more participants than expected re-emerged in the same cluster at T2. In particular, this happened for cluster 3, *p* = 0.02, cluster 4, *p* < 0.01, cluster 7, *p* < 0.001, and cluster 8, *p* < 0.001. Four cells show that more participants than expected re-emerged in a different cluster at T2. This happened, for example, for changes from cluster 2 to cluster 3, *p* = 0.03, for changes from cluster 3 to cluster 5, *p* < 0.01, for changes from cluster 5 to cluster 3, *p* < 0.05, and for changes from cluster 6 to cluster 8, *p* < 0.05. Four cells show that fewer participants than expected re-emerged in a different cluster at T2. This happened for example for changes from cluster 3 to cluster 8, *p* < 0.05, for changes from cluster 7 to cluster 8, *p* < 0.001, for changes from cluster 8 to cluster 3, *p* = 0.02, and for changes from cluster 8 to cluster 7, *p* < 0.001.

These results illustrate that: (a) the majority of participants tended to recognize that all of the three levels of needs described by Maslow’s pyramid should be granted to people with dementia and (b) there is a general trend to “move up” toward the higher level of needs represented in the pyramid from pre- to post-experience.

### Possibility of Becoming Dementia-Friendly

The correlation between the question about the perceived difficulty of taking care of people diagnosed with dementia and the question about perceived knowledge about dementia was explored. Such a correlation was not significant before the ViveDe experience, *r* = 0.13, *n* = 170, *p* = 0.09. The correlation post-experience was significant, *r* = 0.19, *n* = 170, *p* = 0.01. Due to this last correlation, a MANOVA with Time (T1, pre-experience vs. T2, post-experience) as the factor and perceived difficulty and perceived knowledge as dependent variables was run. There was a significant multivariate effect, *F*(2, 168), Wilks’ λ = 53.88, *p* < 0.001. At a univariate level, the effect for knowledge was significant, *F*(1, 169) = 107.64, *p* < 0.001, partial η^2^ = 0.39. Participants increased their perceived knowledge from *M* = 4.71 (SD = 2.08) pre-experience to *M* = 6.06 (SD = 2.08) post-experience. In contrast, the change in perceived difficulty from pre-experience, *M* = 7.94 (SD = 1.66) to post-experience, *M* = 8.04 (SD = 1.64) was not significant, *F*(1, 169) = 0.60, *p* = 0.43, partial η^2^ = 0.00.

## Discussion and Conclusion

The results showed that the ViveDe experience had an impact on how participants considered dementia. Although the idea of needing assistance is not changed by the first-person experience, it significantly reduces the idea that assistance should be delegated to welfare professionals (such as hospitals or nursing homes). We could consider this as a greater positive disposition toward the autonomy of a person with dementia, but we see, however, that this was not confirmed by the analysis. The disposition toward autonomy, though it changes significantly, appears to become more negative. This result is apparently not consistent with the assumptions but can be understood in the light of the firsthand experience provided by ViveDe 360° video.

Precisely because, in an everyday life situation considered to be simple to manage (like buying bread and fruit in a neighborhood shop), the participants felt like they were not able to complete the task without the collaboration of others (the shop managers and the attending customers), a “stereotyped” idea of autonomy materialized from the experience of “frustration” in autonomy. This was to such an extent as to lead participants to change their opinion toward the conception of autonomy to become more restrictive. Therefore, though, on the one hand, this fact confirms the transformative potential of the immersive experience, it also leads us to expand the educational pathways about conceptions of autonomy and the effort required to give assistance to people with dementia within a really inclusive urban community. Indeed, in a Dementia-Friendly perspective, citizens are informed and trained about inclusive behaviors that can best support the autonomy of the person with dementia by avoiding the spontaneous errors of interaction that the participants probably experienced within the video.

This finding appeared to be confirmed by the answers to the knowledge/difficulty questions, in which participants after the experience significantly changed their evaluation of what they knew about dementia but not on the estimated difficulty of being Dementia-Friendly. ViveDe video appears to be successful in being informative about how people with dementia live and how they experience the urban surroundings, but the participants did not receive any new insights into how to supportively interact with and on how to include people with dementia in a daily situation. This suggests the direction in which the interactive dimension of the next ViveDe videos has to be more carefully developed.

Finally, one of the clearest unexpected findings was provided by the positioning on Maslow’s needs pyramid both pre- and post-experience. Not only did the majority of the participants “recognize” that people with dementia have the right to have their basic needs (such as physiological and safety) fulfilled – by highlighting the “welfare” nature of the caregiving relationship – but it appears clear that the participants recognized from the beginning that they also have the higher-level needs (such as social belonging, esteem, and self-actualization) that are generally the needs of any person, regardless of illness. Probably, precisely because the sampling of the participants was on a voluntary basis and the ViveDe experience was proposed indiscriminately to a wide audience, the individuals who participated in the experience did so already thinking that people with dementia have a wide range of needs. In addition, after the ViveDe experience, the urge to consider the needs of a person with dementia appeared even stronger in our participants. In fact, they significantly revised their choices by adding the needs at the higher positions in the pyramid, even when they had not done so previously.

In conclusion, our participants showed they took part in the research with an already assimilated idea of “being a person with dementia” (as advocated by Kitwood’s perspective) and were predisposed, before the ViveDe experience, to be in some way Dementia-Friendly. Moreover, having the opportunity to understand what it could mean to be in that situation by taking a firsthand perspective on dementia enabled participants to revise some opinions about the challenges and skills that an observer generally tends to attribute to a person with dementia when watching a daily interaction from a third-person perspective. This perspective change definitely produced new familiarity with dementia, but it also raised new questions about what daily practices are more suitable to being faced by a person with dementia. Our participants, in fact, significantly modified their understanding of dementia but not their estimation of the difficulties of becoming inclusive toward people with dementia. It must be borne in mind, however, that one important limitation of this study was the lack of a control group and/or a longitudinal measure; hence, the results must be taken with caution. Concerning the former, it is possible that a control group would not be of much help to understand the change of perspective, as control participants would just answer the same questions twice in a very short time-window. As far as the latter, future research should also explore how the change in perspective is affected after longitudinal and repeated exposure to the experience. Future studies could have different aims: to evaluate whether longer exposures to the experience may reinforce its positive effect, as suggested by Zajonc (2001) and to verify whether information that explicitly clashes with one’s previous knowledge can modify previous beliefs (Ecker et al., 2010; Lewandowsky et al., 2012).

Furthermore, the findings presented in this study, even if it could be considered as one of the first significant results in this field, still leave open the question of how to strongly convey a social perspective change that really takes us in the direction of the construction of a stable Dementia-Friendly Community. We acknowledge that ours was a temporally circumscribed intervention, but it had a high experiential and transformative potential. Unfortunately, we cannot measure this change through follow-up monitoring of our participants or explore whether their future actions changed toward being more Dementia-Friendly when they returned home. Thus, although it has shown to be effective, the ViveDe experience still needs a *post hoc* focus on how to convey effective community change that directs toward full inclusion of people with dementia.

## Data Availability Statement

Data access is restricted to protect confidential information about a personal point of view on dementia that can be erroneously considered outside the research context. To access the dataset contact the corresponding author. Data will be available on request with the permission of the third party.

## Ethics Statement

This study involving human participants was reviewed and approved by University of Bergamo. Written informed consent to participate in this study and for the publication of any potentially identifiable images or data included in this article was provided by the participants.

## Author Contributions

FM ideated the research. FM, PS, and NP conducted the experiment. FM and NP wrote the manuscript. FM, NP, and AG collaborated on data analysis. All authors contributed to the article and approved the submitted version.

## Conflict of Interest

The authors declare that the research was conducted in the absence of any commercial or financial relationships that could be construed as a potential conflict of interest.

## Footnotes

1 http://www.vivede.it

2 http://www.homido.com
